# A Pregnancy with Severe Hypertrophic Obstructive Cardiomyopathy after Surgery for an Implantable Cardioverter Defibrillator: A Case Report and Literature Review

**DOI:** 10.1155/2016/4690790

**Published:** 2016-10-18

**Authors:** Takashi Mitsui, Hisashi Masuyama, Kentaro Ejiri, Kei Hayata, Hiroshi Ito, Yuji Hiramatsu

**Affiliations:** ^1^Department of Obstetrics and Gynecology, Okayama University Graduate School of Medicine, Dentistry and Pharmaceutical Sciences, 2-5-1 Shikata, Kita-ku, Okayama 700-8558, Japan; ^2^Department of Cardiovascular Medicine, Okayama University Graduate School of Medicine, Dentistry and Pharmaceutical Sciences, 2-5-1 Shikata, Kita-ku, Okayama 700-8558, Japan

## Abstract

Hypertrophic obstructive cardiomyopathy (HOCM) is cardiac hypertrophy of ventricular myocardium with left ventricular outflow tract obstruction. We report a pregnancy with HOCM after defibrillator implantation surgery. The patient was a 33-year-old nulligravida and was categorized as New York Heart Association class II. Her brain natriuretic peptide (BNP) level was 724.6 pg/dL at preconception. She received careful pregnancy management. However, because frequent uterine contractions were observed at 25 weeks and 6 days of pregnancy, she was hospitalized, and magnesium sulfate was started as a tocolytic agent. At 27 weeks and 5 days of pregnancy, she had respiratory discomfort and orthopnea with a sudden decrease in peripheral oxygen saturation. Cardiac ultrasonography showed a worsened condition of HOCM and her BNP level was 1418.0 pg/mL. We performed an emergent cesarean section and she delivered a boy weighing 999 g. The Apgar score was 8 and 9 points at 1 and 5 minutes, respectively. The mother's heart failure quickly improved after birth and she was discharged at 10 days postoperatively. Fluctuations in circulatory dynamics during pregnancy may sometimes exacerbate heart disease. Therefore, the risks should be fully explained and careful assessment of cardiac function should be performed during pregnancy in patients with severe HOCM.

## 1. Introduction

Hypertrophic cardiomyopathy (HCM) refers to a heart disease where cardiac hypertrophy of the left or right ventricular myocardium occurs without a clear cause of cardiac hypertrophy. Hypertrophic obstructive cardiomyopathy (HOCM) is an uncommon form of cardiomyopathy, which is around 1 in 500 of the general population, that affects the interventricular septum in the area of the left ventricular outflow tract (LVOT). HOCM is characterized by left ventricular (LV) hypertrophy, decreased LV chamber size, and LV diastolic dysfunction [1, 2]. Physiological changes occurring during pregnancy and labor may show or exacerbate symptoms of HOCM [3]. Women of child-bearing age who have a history of syncope are candidates for insertion of an implantable cardioverter defibrillator (ICD) before conception [4, 5].

We report a pregnant woman with severe HOCM after insertion of an ICD who was categorized as New York Heart Association (NYHA) class II before pregnancy.

## 2. Case Presentation

**Figure 1:**
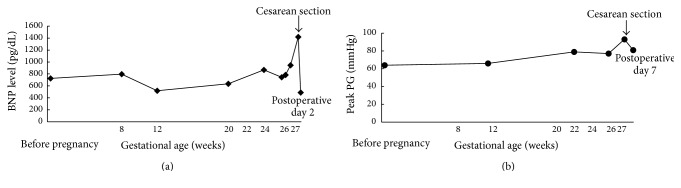
Ontogeny of brain natriuretic peptide levels (a) and the peak pressure gradient of the left ventricular outflow tract (b) during the perinatal period.

The patient was a 33-year-old nulligravida and nullipara. She had a medical history of HOCM, which was diagnosed 1 year ago. She also had a family history of heart disease, where her father suddenly died from dilated cardiomyopathy. In her clinical course, surgery for implanting an ICD was performed because HOCM had been noted approximately 1 year ago and her family history included sudden death due to heart complications. She was treated with oral medication, including 5.0 mg bisoprolol, 30 mg azosemide, 25 mg spironolactone, 300 mg cibenzoline, and 1 mg trichlormethiazide. After a positive pregnancy test, she visited the outpatient office of obstetrics and gynecology, where she was found to be 5 weeks and 6 days pregnant, based on her last menstruation. She was categorized as NYHA class II prior to pregnancy, and her brain natriuretic peptide (BNP) level was 724.6 pg/dL at preconception. Cardiac ultrasonography showed hypertrophy around the full circumference of the LV wall, left ventricular outflow tract obstruction (LVOTO), systolic anterior motion (SAM) of the mitral valve, and mild mitral regurgitation (MR). The peak pressure gradient (PG) of the LVOT was 64 mmHg. When a physician explained that her medical condition and complications might worsen during pregnancy, the patient and her family still decided to continue the pregnancy. Thereafter, she had her pregnancy managed in the outpatient clinic, but at 24 weeks and 6 days of pregnancy, her cervical length was shortened to 22 mm. When frequent uterine contractions were observed on a cardiotocogram at 25 weeks and 6 days of pregnancy, she was hospitalized for management of threatened preterm labor. Her BNP level on admission was 744.3 pg/dL and showed no increase since the first trimester. Magnesium sulfate was administered as a tocolytic agent to suppress the uterine contractions. However, at 26 weeks and 6 days of pregnancy, her BNP level had increased to 945.1 pg/dL, and cardiac ultrasonography showed LVOTO, SAM, and mild to moderate MR. The peak PG of her LVOT was elevated to 77 mmHg. These results indicated that her left atrial and LV pressure was elevated owing to LV diastolic dysfunction. It was thought that her HOCM might be exacerbated by progression of her pregnancy. Her blood magnesium concentrations remained within the range of 6.0 to 6.5 mEq/L, which are effective blood concentrations to suppress uterine contractions. At 27 weeks and 5 days of pregnancy, she suddenly showed respiratory discomfort and wet coughing, accompanied by a decrease in peripheral blood oxygen saturation. Chest radiography showed an enlarged heart shadow and prominent pulmonary congestion. Cardiac ultrasonography showed exacerbated SAM and severe MR, with a peak PG of the LVOT elevated to 93 mmHg. The BNP level was elevated to 1418.0 pg/mL. Continuation of her pregnancy was considered to have worsened the condition of HOCM, causing heart failure and pulmonary edema. We decided that continuing with the pregnancy would potentially involve a serious threat to the mother. Therefore, we performed an emergency cesarean section at 27 weeks and 6 days of pregnancy. She delivered a boy weighing 999 g with an Apgar score of 8 points at 1 minute and 9 points at 5 minutes. He was born without asphyxia and was promptly admitted to the neonatal intensive care unit. Intraoperative blood loss was 415 mL, including amniotic fluid. The mother's heart failure and pulmonary edema markedly improved after delivery. By postoperative day 2, her BNP level was reduced to 487.7 pg/mL (Figure 1(a)). By postoperative day 7, severe MR had improved to mild and the peak PG of the LVOT had improved to 74.6 mmHg (Figure 1(b)). With a favorable postoperative course, she was discharged on postoperative day 10. The newborn was appropriate-for-date, but he was a very-low-birth-weight infant and required artificial respiratory management for respiratory distress syndrome. Grades I and II of intraventricular hemorrhage were observed at 4 days after birth. However, a magnetic resonance imaging scan at 91 days after birth showed no changes indicating hydrocephalus or cerebral hemorrhage. Myelination abnormalities, such as periventricular leukomalacia, were not observed. At the time of writing this report, cerebroneurological disorders or developmental disorders have not been observed.

## 3. Discussion

Pregnant women complicated by HCM are frequently hospitalized because of a cardiovascular event [6]. HCM is often resistant to therapy if complicated by heart failure, arrhythmia, and other complications [7]. Our patient had a family history of sudden death from DCM in her father. Some cases of HCM have been known to transition to a pathology of DCM and are referred to as a dilated phase of HCM. A dilated phase of HCM is also called end-stage HCM, where there is the potential for the condition to suddenly worsen. Because the present case also included a family history of sudden death, the patient underwent surgery to implant an ICD. We also needed to carefully consider whether continuing her pregnancy and giving birth were appropriate. Boule et al. [8] reported a study of 20 cases of pregnancy after surgery for implanting an ICD in which the ICD worked without problems during pregnancy. They also did not find any ICD-related adverse events during pregnancy [8]. Therefore, pregnancy is regarded as being fundamentally possible after ICD implantation in cases of HCM. However, more careful and appropriate management is needed because of worsening of disease or the complication of arrhythmia during pregnancy and after birth.

HOCM includes morphological features, such as asymmetric septal hypertrophy and SAM. Failure of the mitral valve to close due to SAM may result in MR [9]. SAM-associated obstruction to LV outflow and MR may result in a decrease of cardiac output and lead to symptomatic heart failure which may be exacerbated during pregnancy due to a decrease in vascular resistance and/or an increase in circulating plasma volume. These changes during pregnancy can cause an increase in MR and in the PG of the LVOT [10]. In the present case, the BNP level was 744.3 pg/mL at the time of admission at 25 weeks and 6 days of pregnancy, and it did not show a clear upward trend from the first trimester. However, on cardiac ultrasound at 1 week after admission, the peak PG of the LVOT had risen to 79 mmHg, and MR had worsened from mild to moderate. Fluctuation in circulatory dynamics caused by pregnancy might have exacerbated the patient's condition of HOCM.

Our patient also had shortening of the cervical length and developed frequent uterine contractions. These conditions required treatment for threatened preterm labor. Magnesium sulfate was used as a tocolytic agent for our patient. Magnesium sulfate inhibits muscle contractions by inhibiting the influx of calcium ions from the cell exterior to the cell interior. Once magnesium is within the cell, magnesium inhibits the release of calcium from the endoplasmic reticulum. The myocardium and vascular smooth muscle have *β*-receptor blocking activity, vascular smooth muscle relaxant activity, antiarrhythmic activity, and a myocardial protective activity after ischemia. In the present case, vascular smooth muscle relaxant activity of magnesium sulfate might have been one of the causes that provoked a further increase in the PG of the LVOT and a worsening of MR. Consequently, this could have caused our patient to develop heart failure and pulmonary edema. Other tocolytic agents include ritodrine hydrochloride and calcium antagonists, but ritodrine hydrochloride could not be used because our patient was taking an oral *β*-blocker. With regard to calcium antagonists, nifedipine is increasingly being considered as an option for treating threatened preterm labor [11]. We investigated use of nifedipine in the present case. However, some studies have reported that nifedipine can worsen the PG of the LVOT by its peripheral vasodilatory activity [12]. Therefore, we did not use nifedipine.

Vaginal delivery is typically considered possible for patients with HCM and HOCM [13]. We considered vaginal delivery under epidural anesthesia in the present case, but at 27 weeks and 6 days of pregnancy, she had heart failure and pulmonary edema. Therefore, we decided to perform emergency cesarean section owing to the urgent need to rapidly improve the mother's circulatory dynamics. Tanaka et al. reported cardiovascular events in pregnancy with HCM [14].  Table 1 shows cases with HOCM in their report [14]. Our patient was categorized as NYHA class II prior to pregnancy, and cardiac ultrasonography showed mild MR and the peak PG of LVOTO was 64 mmHg. Case 4 shown in Table 1 had a similar condition to our case regarding NYHA class II, MR, and a high peak PG of LVOTO. In patients with a poor condition of HOCM from prepregnancy, termination of pregnancy might be considered in the early weeks of pregnancy because of exacerbation of the maternal circulation. Cesarean section for our patient was performed under lumbar anesthesia combined with epidural anesthesia. Heart failure and pulmonary edema in our patient markedly improved after she gave birth. Prompt termination of pregnancy may have prevented severe complications in the mother or an irreversible deterioration in cardiac function.

In a pregnancy complicated by HOCM with a family history of sudden death or a pregnancy associated with a high degree of LVOTO, fluctuations in circulatory dynamics caused by pregnancy may sometimes exacerbate the underlying disease. Therefore, if pregnancy is continued, there needs to be a full explanation of the risks, as well as careful assessment of cardiac function, throughout the duration of pregnancy.

## Figures and Tables

**Table 1:** Pregnancies complicated by hypertrophic obstructive cardiomyopathy (from [14]).

Case	Age	Delivery methods	Indication of C/S	Delivery weeks	Medication at preconception	NYHA class at preconception	NYHA class during pregnancy	NYHA class after birth	MR (≧moderate)	LVOTO (>50 mmHg)
1	25	Vaginal delivery		38	Metoprolol, verapamil	1	1	1	−	−
2	32	Cesarean section	PH and elevated PG of LVOTO	31	Verapamil	1	1	1	−	−
3	39	Vaginal delivery		40	None	1	1	1	−	−
4	30	Cesarean section	Elevated PG of LVOTO	27	Diltiazem	2	3	2	+	+
5	25	Cesarean section	Nonsustained ventricular tachycardia	29	Mexiletine, metoprolol	1	1	1	−	−
6	30	Cesarean section	Previous cesarean section	37	Mexiletine, metoprolol	1	1	1	−	−
7	32	Vaginal delivery		37	None	1	1	1	−	−

PH: pulmonary hypertension, PG: pressure gradient, LVOT: left ventricular outflow tract, NYHA: New York Heart Association, and MR: mitral regurgitation.
